# Farnesoid X Receptor Induces Murine Scavenger Receptor Class B Type I via Intron Binding

**DOI:** 10.1371/journal.pone.0035895

**Published:** 2012-04-23

**Authors:** Guodong Li, Ann M. Thomas, Jessica A. Williams, Bo Kong, Jie Liu, Yuka Inaba, Wen Xie, Grace L. Guo

**Affiliations:** 1 Department of Pharmacology, Toxicology and Therapeutics, University of Kansas Medical Center, Kansas City, Kansas, United States of America; 2 Department of Abdominal Surgery, Cancer Treatment Center, The Fourth Affiliated Hospital of Harbin Medical University, Harbin, People's Republic of China; 3 Center for Pharmacogenetics and Department of Pharmaceutical Sciences, University of Pittsburgh, Pittsburgh, Pennsylvania, United States of America; University of Bari & Consorzio Mario Negri Sud, Italy

## Abstract

Farnesoid X receptor (FXR) is a nuclear receptor and a key regulator of liver cholesterol and triglyceride homeostasis. Scavenger receptor class B type I (SR-BI) is critical for reverse cholesterol transport (RCT) by transporting high-density lipoprotein (HDL) into liver. FXR induces SR-BI, however, the underlying molecular mechanism of this induction is not known. The current study confirmed induction of SR-BI mRNA by activated FXR in mouse livers, a human hepatoma cell line, and primary human hepatocytes. Genome-wide FXR binding analysis in mouse livers identified 4 putative FXR response elements in the form of inverse repeat separated by one nucleotide (IR1) at the first intron and 1 IR1 at the downstream of the mouse *Sr-bi* gene. ChIP-qPCR analysis revealed FXR binding to only the intronic IR1s, but not the downstream one. Luciferase assays and site-directed mutagenesis further showed that 3 out of 4 IR1s were able to activate gene transcription. A 16-week high-fat diet (HFD) feeding in mice increased hepatic *Sr-bi* gene expression in a FXR-dependent manner. In addition, FXR bound to the 3 *bona fide* IR1s *in vivo*, which was increased following HFD feeding. Serum total and HDL cholesterol levels were increased in FXR knockout mice fed the HFD, compared to wild-type mice. In conclusion, the *Sr-bi*/SR-BI gene is confirmed as a FXR target gene in both mice and humans, and at least in mice, induction of *Sr-bi* by FXR is via binding to intronic IR1s. This study suggests that FXR may serve as a promising molecular target for increasing reverse cholesterol transport.

## Introduction

FXR (farnesoid X receptor, NR1H4) is a bile acid-activated transcription factor and a member of the nuclear receptor superfamily. Strongly expressed in the liver and intestine, FXR has been shown to be the master transcriptional regulator not only of the biosynthesis and enterohepatic circulation of bile acids, but also of cholesterol and triglyceride homeostasis [1], [2], [3], [4]. Disruption of the FXR gene in mice results in a variety of pathophysiological conditions, including a proatherosclerotic lipid profile with increased serum cholesterols and triglycerides [1], cholestasis, non-alcoholic fatty liver diseases, cholesterol gallstone disease, hepatocellular carcinoma, and intestinal inflammation and tumors [5], [6].

Scavenger receptor class B type I (SR-BI) is a cell surface glycoprotein and was first cloned in 1994 as the receptor mediating selective uptake of high-density lipoprotein (HDL)-cholesterol into liver, adrenals, testes, and ovaries [7], [8], [9], [10]. As a HDL receptor, SR-BI is a key regulator in enhancing reverse cholesterol transport (RCT) in the liver, and hepatic over-expression of SR-BI can decrease plasma levels of HDL cholesterol, which may have anti-atherosclerosis effects [10], [11], [12]. One of the mechanisms by which FXR is involved in regulating cholesterol and bile acid homeostasis is via transcriptional regulation of target gene expression. FXR has previously been shown to induce SR-BI expression [4], [13], [14]. However, the underlying molecular mechanism by which FXR induces SR-BI expression is not fully defined. Therefore, the purpose of the current study is to determine the molecular mechanism by which FXR regulates SR-BI expression in human and mouse models.

## Results

### Activation of FXR induced SR-BI expression in mouse livers, primary human hepatocytes and human hepatoma cell line, HepG2 cells

**Figure 1:**
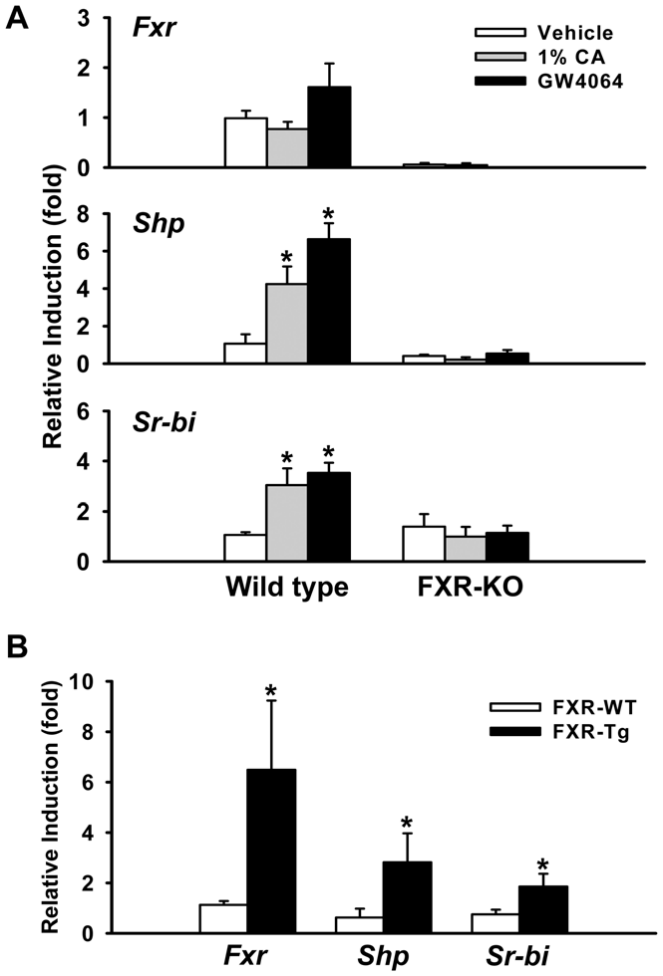
*Sr-bi* transactivation is FXR dependent in mouse liver. **A**, Induction of *Sr-bi* mRNA expression in the liver following treatment with either 1% cholic acid (CA)-containing diet or GW4064 in WT and FXR-KO mice as described in the Methods. *Shp* gene expression level serves as a positive control to indicate FXR activation. An asterisk indicates P<0.05 between vehicle and ligand treatment group. **B**, *Fxr*, *Shp* and *Sr-bi* mRNA expression levels in liver of FXR-WT and FXR-Tg mice. An asterisk means P<0.05 between FXR-WT and FXR-Tg group.

First, the induction of hepatic *Sr-bi* was determined in mice treated with FXR natural and synthetic ligands, cholic acid (CA) and GW4064, respectively, as well as by genetic over-expression of FXR (FXR-Tg mice). The activation of FXR was first verified by determining the mRNA expression of bona fide FXR targets [15], [16]. Strong induction of small heterodimer partner (*Shp*), organic solute transporter β (*Ostβ*), and bile salt export pump (*Bsep*), as well as great suppression of cytochrome P450, family 7, subfamily A, polypeptide 1 (*Cyp7a1*) and Na+/taurocholate cotransporting polypeptide (*Ntcp*), were shown in livers of mice either treated with FXR agonist or with transgenic over-expression of FXR (**Figure 1**, **Figure S1**). The induction of *Sr-bi* was then determined and results showed activation of FXR induced *Sr-bi* mRNA 3.0, 3.5, and 2.8 fold with CA, GW4064 and transgenic expression of FXR, respectively (**Figure 1A, B**). Furthermore, the induction appears to be FXR-mediated because FXR-knockout (KO) mice did not have increased *Sr-bi* expression following CA or GW4064 treatment.

**Figure 2:**
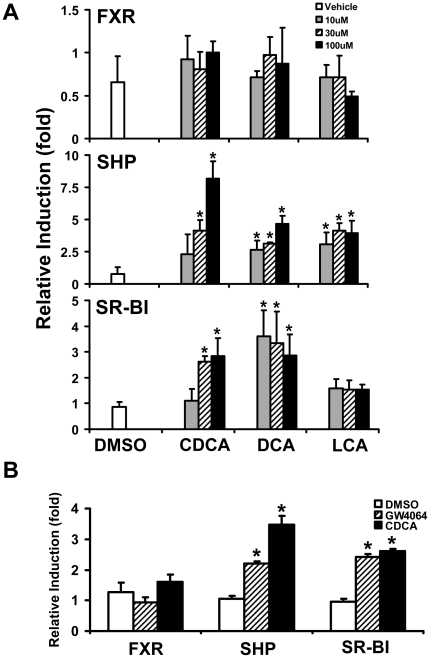
FXR activation induces SR-BI gene expression in primary human hepatocytes and HepG2 cells. **A**, Primary human heptaocytes were treated with 0.1% DMSO as a negative control or increasing concentrations of CDCA, DAC and LCA (10, 30 and 100 µM) for 48 hrs. The mRNA levels of FXR, SHP, and SR-BI were determined by Q-PCR. An asterisk means P<0.05 between cholic acid treatment and control group. **B**, HepG2 cells were treated with 0.1% DMSO as a negative control, 500 nM GW4064 or 100 µM CDCA for 24 hrs, respectively. The mRNA levels of FXR, SHP, and SR-BI were determined by Q-PCR. An asterisk indicates P<0.05 between FXR ligand treatment and no treatment control group.

To further test whether SR-BI is a FXR target gene in humans, the induction of SR-BI in primary human hepatocytes and in HepG2 cells was determined. In primary human hepatocytes, SHP mRNA levels were induced by increasing concentrations of chenodeoxycholic acid (CDCA), deoxycholic acid (DCA), and lithocholic acid (LCA), but the SR-BI mRNA levels were induced only by increasing concentrations of CDCA and DCA, but not LCA (**Figure 2A**). In addition, FXR activation by GW4064 or CDCA treatment increased mRNA levels of SR-BI in HepG2 cells, which are commonly used as a substitute for human hepatocytes (**Figure 2B**).

### FXR binds to multiple regions in the mouse *Sr-bi* gene

**Figure 3:**
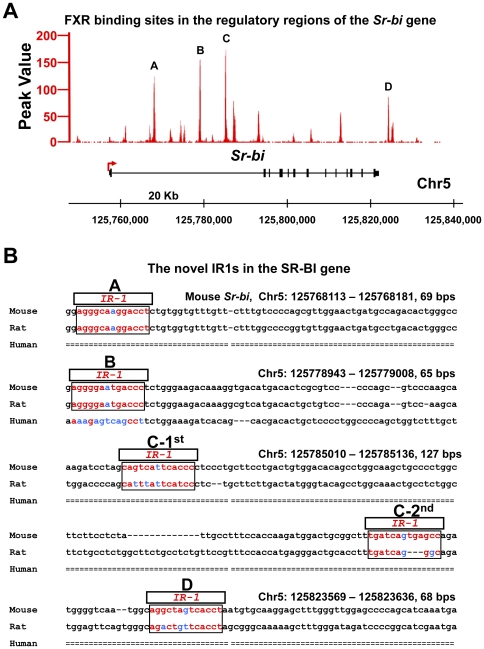
Novel FXR response elements, IR1s, in the first intron and downstream of the mouse *Sr-bi* gene. **A**, The novel four FXR binding sites (A, B, C, and D) in the *Sr-bi* gene identified by ChIP-seq analysis. **B**, The novel IR1s (A, B, C-1^st^, C-2^nd^ and D) identified at the first intron and downstream of the *Sr-bi* gene are conserved within mouse and rat, but not human, *Sr-bi*/SR-BI gene. The novel IR1s are marked in the box.

The mechanism of induction of SR-BI by FXR in humans has been shown to be a result of direct binding of FXR to a direct repeat separated by 8 nucleotides (DR8) response element in the promoter of the SR-BI gene [14]. However, the mechanism of SR-BI induction in mice is not known, and it is necessary to determine species differences in order to use mouse models to study the regulation of SR-BI in humans. According to the published ChIP-seq (chromatin immunoprecipitation coupled with high-throughput DNA sequencing) analysis [15], [17], FXR does not bind to promoter regions of the mouse *Sr-bi* gene. Instead, novel FXR binding sites were indentified in three regions within the first intron (A, B and C) and the downstream region (D) of the *Sr-bi* gene (**Figure 3A**). Further analysis showed that all FXR binding regions contained putative FXR response elements (FXRREs) in the form of inverted repeats separated by one nucleotide (IR1) (**Figure 3B**). Moreover, binding region C contained two IR1s (**Figure 3B**). The sequence of these novel IR1s was shown to be highly conserved in mice and rats, but not in humans (**Figure 3B**).

**Figure 4:**
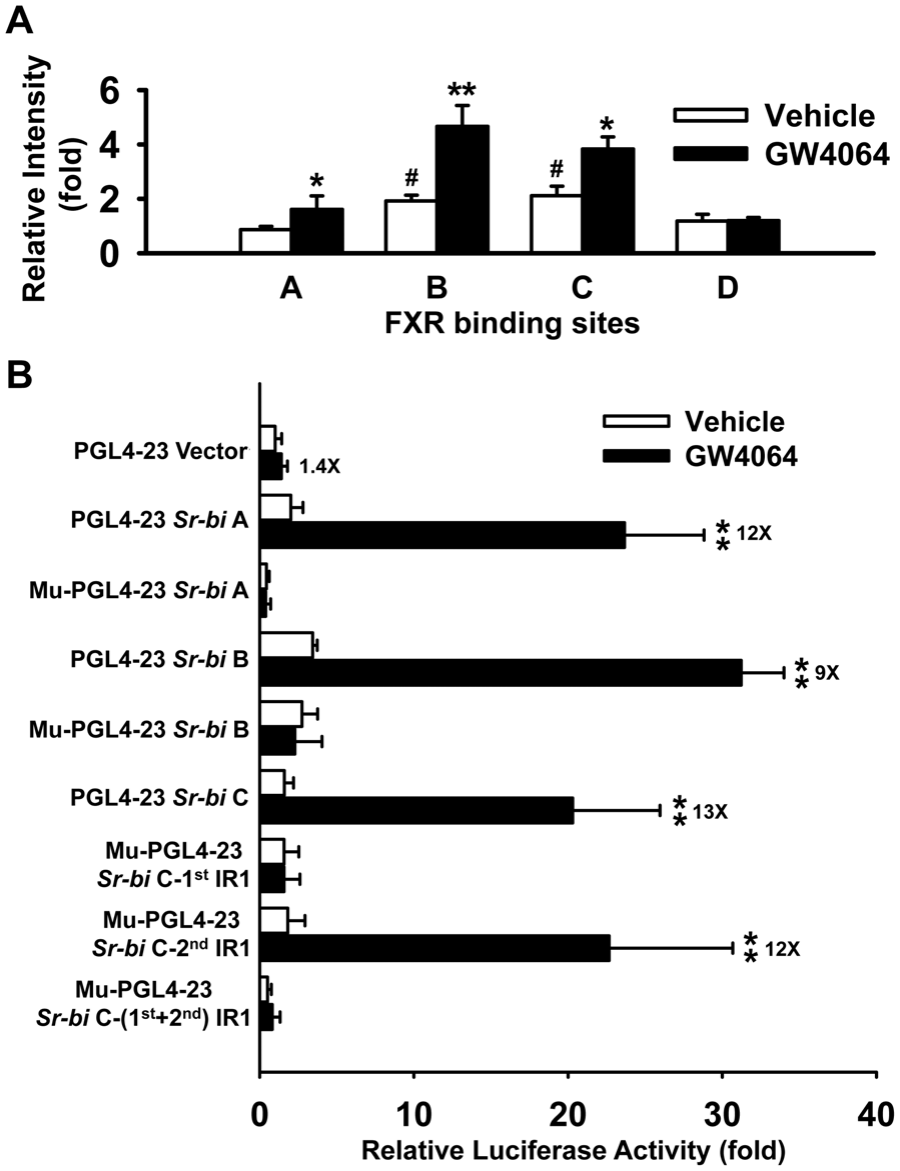
FXR activates *Sr-bi* by binding to the IR1s within the first intron, but not the downstream region of the *Sr-bi* gene. **A**, ChIP-qPCR assays showed binding of FXR to the intron (binding sites A, B, and C) but not the downstream region (binding sites D) of *Sr-bi* gene containing the novel IR1s in mouse liver after treatment with GW4064. The relative intensity (fold) indicates fold increase over vehicle treatment. n = 5 mice per group. A pound sign indicates P<0.05 between binding site A and other binding sites. An asterisk means P<0.05 and double asterisks mean P<0.01 between vehicle and GW4064 treatment. **B**, Luciferase assay revealed that, upon FXR activation, the IR1s at the first intron of *Sr-bi* gene are functional in enhancing gene transcription. The DNA constructs containing the novel FXR binding sites (IR1s) found in the first intron of *Sr-bi* gene and the DNA constructs containing mutant corresponding novel IR1s were cloned into a pGL4-23 firefly luciferase vector and transfected into HepG2 cells as indicated in the Methods. The cells were then treated with vehicle (0.1% DMSO) or 1 µM GW4064 for 36 hrs followed by evaluation of luciferase activity. The white bar is for cells treated with vehicle and the black bar is for cells treated with 1 µM GW4064. The relative luciferase activity (fold) indicates fold increase over empty vector by vehicle treatment. The fold induction of luciferase activity by these constructs with GW4064 treatment compared to DMSO is indicated next to the black bars. Double asterisks indicate P<0.01 between vehicle and GW4064 treatment.

To validate FXR binding to the novel IR1s identified in the *Sr-bi* gene, ChIP-qPCR (chromatin immunoprecipitation quantitative real-time PCR) was performed on livers of mice treated with vehicle or GW4064. In mice treated with vehicle, FXR bound to regions B and C. In mice treated with GW4064, FXR binding increased within all three of these regions in mouse livers (**Figure 4A**). However, FXR did not bind to site D located downstream of the *Sr-bi* gene in livers of either vehicle or GW4064 treated mice (**Figure 4A**).

### Functional assessment of the novel FXR binding sites in the *Sr-bi* gene by luciferase reporter gene assay and site-directed mutagenesis assay

Luciferase reporter gene assays were used to determine whether the three FXR binding sites (A, B and C) in the *Sr-bi* gene are functional in enhancing transcriptional activity. Compared to the vehicle control, all three binding sites in the first intron of the *Sr-bi* gene (A, B and C) were effective in inducing luciferase activity 12, 9 and 13 fold, respectively (**Figure 4B**). The transcriptional activations were greatly diminished, when the IR1 sequences were mutated in binding sites A and B of the *Sr-bi* gene. The luciferase gene expression was significantly decreased after mutation of the first IR1 sequences alone or of both IR1 sequences in binding site C. However, the transcriptional activation was not affected by mutation of the second IR1 sequences alone in binding site C (**Figure 4B**).

### Serum cholesterol profiles and hepatic total cholesterol levels were increased in FXR-KO mice

**Figure 5:**
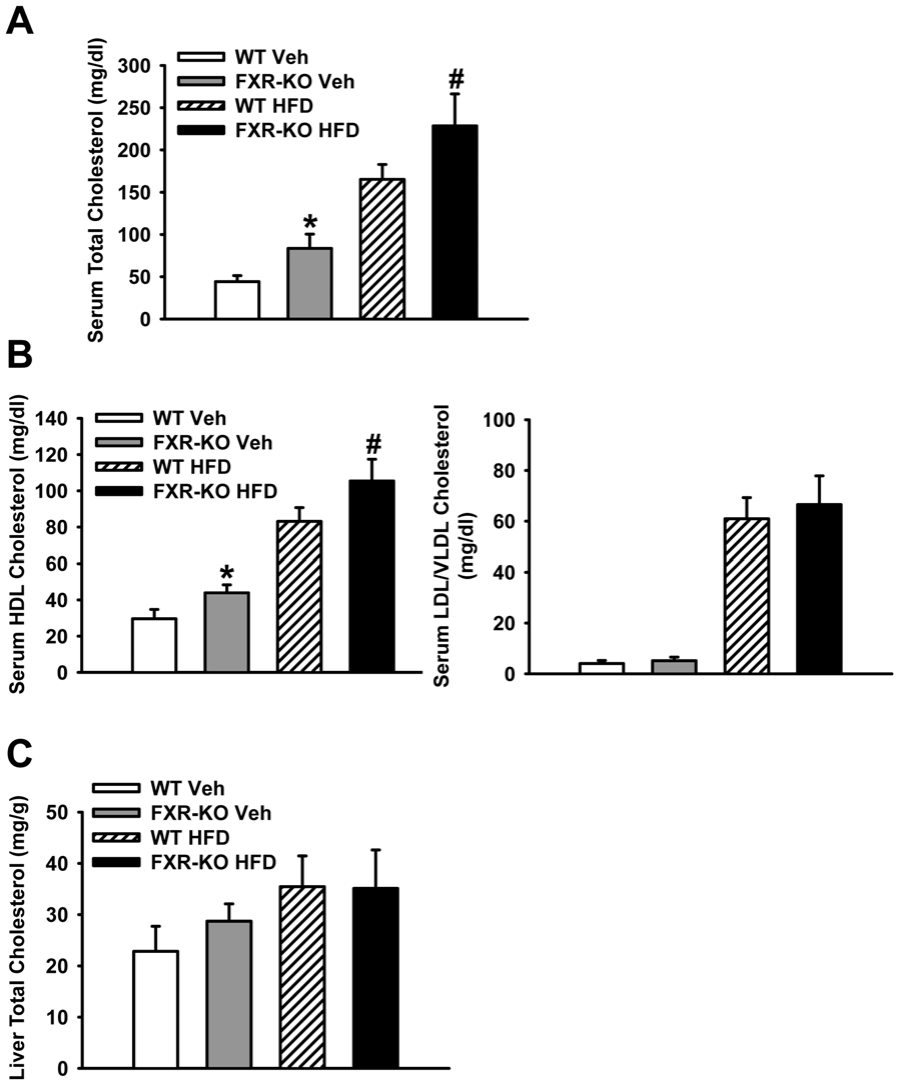
Cholesterol profile of wild type and FXR-KO mice fed the control or high fat-containing diet. Serum and liver lipids were isolated from WT and FXR-KO mice treated with vehicle or high fat-containing diet (HFD) for 16 weeks. Serum total cholesterol (**A**), serum HDL and LDL/VLDL cholesterol (**B**), as well as liver total cholesterol (**C**) were measured as described in the Methods. n = 5–7 mice per group. An asterisk means P<0.05 between WT and FXR-KO vehicle group. A pound indicates P<0.05 between WT and FXR-KO HFD group.

To further determine the physiological consequences of FXR induction of *Sr-bi* on HDL transport into the liver, serum cholesterol profiles and hepatic total cholesterol levels were measured in FXR-KO and wild-type (WT) mice fed a control or high-fat diet (HFD) for 16 weeks. Serum total cholesterol level increased in FXR-KO mice when compared to WT mice regardless of diet type (**Figure 5A**). Further analysis showed that the increased serum cholesterol level in FXR-KO mice was due to increase in HDL cholesterol but not in LDL/VLDL cholesterol (**Figure 5B**). Even though the total hepatic cholesterol level slightly increased in FXR-KO mice fed a control diet, the hepatic cholesterol level did not significantly change between WT and FXR-KO mice fed a HFD (**Figure 5C**).

### Activation of FXR by HFD treatment induced *Sr-bi* gene expression in WT mouse liver by direct binding of FXR to multiple sites in the *Sr-bi* gene

**Figure 6:**
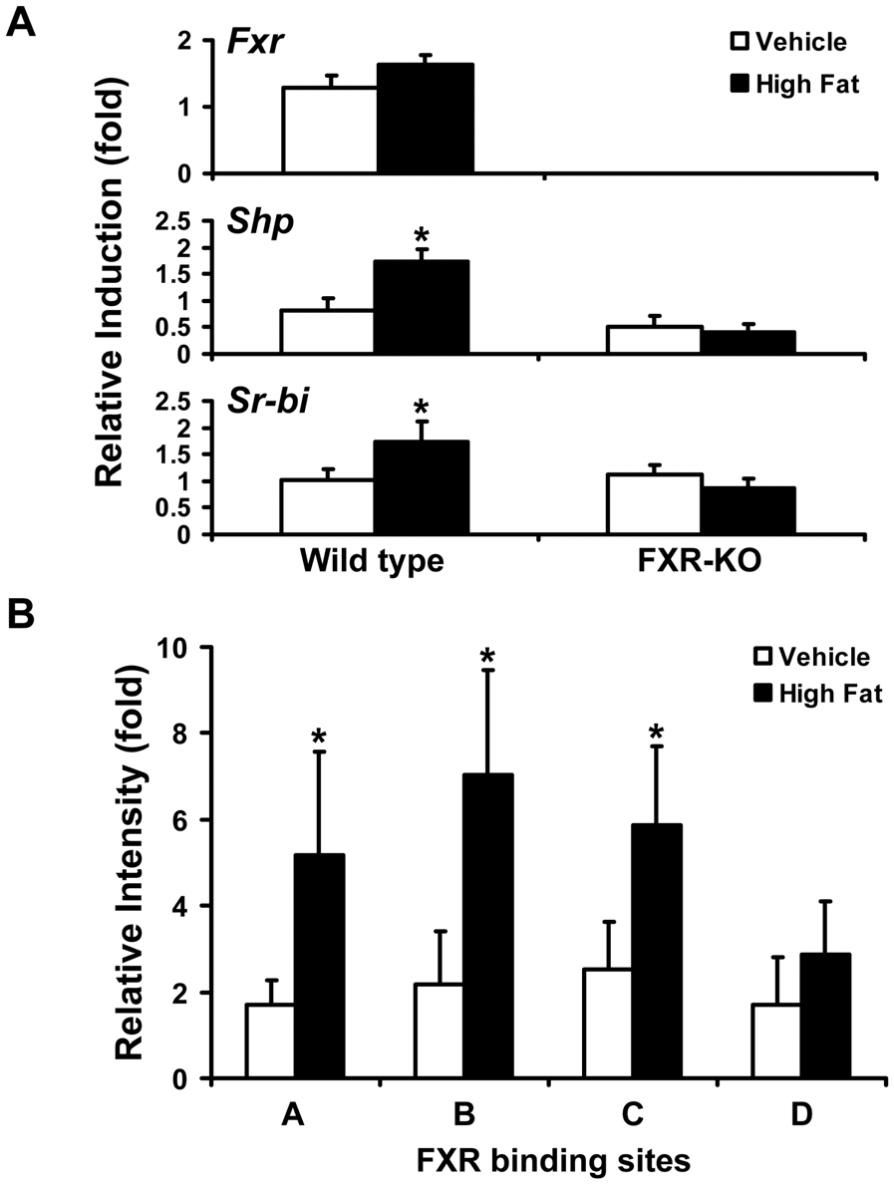
FXR activation by HFD treatment increases *Sr-bi* expression by direct binding of FXR to multiple FXR binding sites in the *Sr-bi* gene. **A**, Induction of *Sr-bi* mRNA expression in liver following treatment with vehicle or high fat-containing diet (HFD) in WT and FXR-KO mice, as described in the Methods. Hepatic mRNA levels of *Fxr*, *Shp*, and *Sr-bi* were determined by Q-PCR. n = 5 mice per group. An asterisk means P<0.05 between treatment and vehicle control group. **B**, ChIP-qPCR assays show binding of FXR to the first intron but not the downstream region containing the novel IR1s in the *Sr-bi* gene in mouse livers after treatment with HFD. The relative intensity (fold) indicates fold increase over vehicle treatment level. n = 4 mice per group. An asterisk indicates P<0.05 between vehicle and HFD treatment group.

FXR activation in liver by HFD feeding was evaluated in WT and FXR-KO mice. HFD, administered for 16 weeks, induced the mRNA levels of *Fxr* by 1.5 fold, *Shp* by 1.8 fold and *Sr-bi* by 1.6 fold in the livers of WT mice, but not in FXR-KO mice (**Figure 6A**). Furthermore, ChIP-qPCR assays showed that HFD treatment, similar to GW4064, increased the binding of FXR to the three binding sites (A, B and C) in the first intron of the *Sr-bi* gene (**Figure 6B**). However, FXR did not bind to the downstream region (site D) of the *Sr-bi* gene by HFD treatment (**Figure 6B**).

## Discussion

The present study showed that SR-BI mRNA was induced in a FXR-dependent manner in mouse livers, primary human hepatocytes and HepG2 cells. Our results suggest that FXR regulates *Sr-bi* gene expression by binding to multiple IR1s in the first intron of the *Sr-bi* gene. Moreover, the serum HDL level was increased in FXR-KO mice when fed either a control or HFD. Increased *Sr-bi* mRNA levels were shown to be FXR-dependent under HFD feeding by direct binding of FXR to the first intron of the *Sr-bi* gene, which indicates that FXR may enhance HDL uptake into the liver via inducing *Sr-bi* gene transcription.

Activation of FXR has been demonstrated to regulate the expression of many hepatic genes involved in lipid homeostasis, including SR-BI [4]. Consistent with our findings (**Figure 1** **,** **2**), *Sr-bi* mRNA levels have been shown to increase in livers of C57BL/6J mice but not in livers of FXR-KO mice upon LCA and CA feeding [4], [18], [19]. However, a conflict result has been reported that *Sr-bi* expression was reduced following administration of CDCA or GW404764 (a FXR agonist) both *in vivo* and *in vitro*. The authors further demonstrated that the decrease of *Sr-bi* was mediated by the FXR/RXR-SHP-liver receptor homologue 1 (LRH-1) pathway [20]. These conflicting results may be due to the different experimental models and/or ligands used.

The underlying molecular mechanisms of FXR in regulating SR-BI and the role of SR-BI in FXR-mediated lipid homeostasis are still not clear. Recently, a vast database of nuclear receptor binding sites has been established with the development of genome-wide discovery of transcription factor binding sites by ChIP-on-chip (chromatin immunoprecipitation coupled to microarray technology) and ChIP-seq techniques [21], [22], [23]. These data have shown that transcription factors tend to bind to multiple sites in the promoter and/or enhancer regions of target genes [17], [24], [25], [26]. Although our original ChIP-seq data did not show FXR binding to the promoter region of *Sr-bi* (**Figure 3A**), we found three FXR binding sites within the first intron of *Sr-bi* gene (**Figure 3A, B**). Moreover, even though luciferase activity did not correlate with the abundance of FXR binding, all three of these novel response elements, which include IR1s, were demonstrated functional (**Figure 4**). Site-directed mutagenesis results further demonstrated that the IR1s in binding sites A and B, as well as the first but not the second IR1 in binding site C are functional for FXR regulation of *Sr-bi* gene expression. These results indicate that activation of FXR could induce *Sr-bi* transcription by directly binding to multiple IR1s located in *Sr-bi* gene. However, a recent study showed that FXR up-regulated *Sr-bi* in mouse hepatocytes through a FXR-pJNK-hepatocyte nuclear factor 4 α (HNF4 α)-SR-BI pathway, which indicates FXR may regulate SR-BI in both direct and indirect manners [13]. In addition to FXR, another nuclear receptor, peroxisome proliferator-activated receptor α (PPARα), can also increase *Sr-bi* expression in liver of rats [27]. PPARα has also been shown to be activated by FXR in HepG2 cells [28], which may represent another indirect mechanism of FXR in induction of *Sr-bi* expression in human. However, activation of PPARα in mouse livers by fibrates decreased hepatic *Sr-bi* protein expression without changing *Sr-bi* mRNA levels. The posttranscriptional regulatory effect of fibrates on murine hepatic *Sr-bi* protein levels was further demonstrated PPARα dependent using PPARα deficient mice [29]. These controversial results on *Sr-bi* regulation by activation of PPARα may due to species-specific differences.

Even though no IR1 was found in the human SR-BI gene compared to the mouse gene (**Figure 3B**), our results still showed that activating FXR increased SR-BI expression in primary human hepatocytes and a human hepatoma cell line (**Figure 2**). One recent study demonstrated that FXR directly activates SR-BI gene transcription by binding to a DR8 motif in the promoter region of the human SR-BI gene [14], which may help in understanding the underlying molecular mechanisms of FXR in regulating human SR-BI expression. Increasing studies also have shown that a variety of nuclear receptors, including liver X receptors (LXR), LRH-1, peroxisome proliferator-activated receptor γ (PPARγ), and HNF4α can stimulate hepatic SR-BI gene expression in humans [30], [31], [32], [33]. Thus, FXR may also modulate SR-BI expression through regulating or interacting with these nuclear receptors or signaling pathways. These data suggest that activation of FXR could up-regulate hepatic SR-BI transcription either directly or through coordinating the activity of other nuclear receptors in both mouse and human livers.

Hepatic SR-BI has been shown to serve as a key mediator of RCT by taking of HDL cholesterol to the liver [34]. A series of studies using transgenic or recombinant adenovirus-mediated mice showed that hepatic over-expression of SR-BI markedly reduces atherosclerosis [10], [11], [35]. Furthermore, SR-BI KO mice have higher HDL cholesterol in the circulation and enhanced atherosclerosis development [36]. These results suggest that hepatic SR-BI is critical in protecting against atherosclerosis development. Our studies showed that, compared to WT mice, FXR-KO mice had more serum total and HDL cholesterol (**Figure 5**). Since SR-BI plays a key role in mediating selective HDL cholesterol uptake in the liver, these results indicate that the high levels of serum total and HDL cholesterol in FXR-KO mice may at least be partially due to reduction of *Sr-bi* expression. Our results also found that HFD induced hepatic *Sr-bi* expression (**Figure 6A**) and this induction was, at least partially, by increasing FXR binding to multiple IR1s in the first intron of the *Sr-bi* gene (**Figure 6B**). Thus, the accumulation of HDL cholesterol in the circulation of FXR-KO mice was at least partially due to the loss of FXR regulation of *Sr-bi* expression. These combined findings further established that FXR is a physiological modulator of SR-BI which may enhance HDL reverse cholesterol transport. Thus, induction of SR-BI by activation of FXR may help prevent atherosclerosis. These findings, together with the recent finding that FXR agonists protect against atherosclerosis [37], [38], [39], suggest that FXR is a potential therapeutic target for maintaining cholesterol homeostasis as well as for treatment of hypercholesterolemia and coronary heart disease.

In summary, the current study identified *Sr-bi*/SR-BI as a FXR target gene in both mouse and human livers. The molecular mechanism of FXR regulation of *Sr-bi* gene expression is via direct binding of FXR to multiple novel FXRREs in the first intron of the *Sr-bi* gene. Increased total and HDL cholesterol in FXR KO mice may, at least partly, due to reduced *Sr-bi* expression.

## Materials and Methods

### Animals and treatments

WT and FXR-KO male mice in C57BL/6J genetic background were used in this study (8–10 weeks old, n = 4–6 per group). FXR-KO mice have been backcrossed with C57BL/6J mice for over 10 generations and were confirmed with >99.99% C57BL/6J background. The VP-FXR transgenic mice were created by over-expressing constitutively active FXR (VP-FXR) in the liver and intestine using the tetracycline-inducible transgenic system. The VP-FXR transgenic mice were DOX positive; therefore, the DOX negative mice were used as controls. All mice were housed in pathogen-free animal facilities under a standard 12-h light/dark cycle with free access to food and autoclaved tap water. The control and HFD were obtained from LabDiet (Olathe, KS) and Jackson Laboratories (Bar Harbor, ME), respectively. The control diet (5015) contained 17.8% protein, 64.8% carbohydrate, and 5.8% fat. The HFD (D12492) contained 26.2% protein, 26.3% carbohydrate, and 34.9% saturated fat. The studies were carried out in strict accordance with the recommendations in the Guide for the Care and Use of Laboratory Animals of the National Institutes of Health. All protocols and procedures were approved by the University of Kansas Medical Center Animal Care and Use Committee. The protocol number approved for these studies was 2010–1947.

To determine *Sr-bi* mRNA levels in liver by quantitative PCR (qPCR), mice were fed with 1% (w/w) CA-containing diet or regular diet for 5 days. The CA diet was made in house by mixing CA (Sigma, St. Louis, MO) with regular wet rodent chow diet, which was then completely dried. To determine *Sr-bi* mRNA induction and FXR binding to the intron and downstream of the *Sr-bi* gene by ChIP-qPCR, mice were orally gavaged with 75 mg/kg GW4064 or vehicle twice (first dosage at 6 pm and second dosage at 8 am next day) with livers harvested 4 hrs after the second treatment. GW4064, which was synthesized by the Chemical Discovery laboratory at the University of Kansas (Lawrence, KS), was dissolved in vehicle (PBS containing 1% methylcellulose and 1% Triton-100). For HFD feeding study, mice were fed either a regular rodent chow or the HFD for 16 weeks. At the end of the feeding, mice were subjected to fasting overnight before tissue collection. Mouse livers were quickly removed, snap-frozen in liquid nitrogen and stored at −80°C until use.

### Cell culture

A human hepatocellular carcinoma cell line, HepG2, was purchased from the American Type Culture Collection (Manassas, VA). Primary human hepatocytes were obtained from the University of Pittsburgh through the Liver Tissue Cell Distribution System (NIH Contract #N01-DK-7-0004/HHSN267200700004C). The primary human hepatocytes were obtained by written consents from the participants. The research on primary human hepatocytes has been approved by the institutional review board in the University of Kansas Medical Center and University of Pittsburgh. HepG2 cells were cultured in high-glucose DMEM supplemented with 1% penicillin/streptomycin, 1% L-glutamine, and 10% fetal bovine serum (Omega Scientific, Tarzana, CA). Primary human hepatocyte culture was performed following published methods [40]. All cells were maintained in 5% CO_2_ humidified atmosphere at 37°C. For treatment with bile acids (CDCA, DCA, or LCA) or GW4064 in primary human hepatocytes, the chemicals were dissolved in DMSO and diluted to 10 µM, 30 µM, and 100 µM in cell culture medium before treating cells for 48 hrs for measurement of gene expression. HepG2 cells were also treated with 100 µM CDCA or 500 nM GW4064 for 24 hrs for measurement of gene expression.

### RNA isolation and quantitative real-time PCR (Q-PCR)

Total RNA was isolated from cells and frozen livers using Trizol reagent (Sigma, Saint Louis, MO) according to the manufacturer's instructions. The concentration of total RNA was determined by spectrophotometry with the integrity confirmed by MOPS gel electrophoresis. The mRNA expression levels of FXR/*Fxr*, SHP/*Shp*, SR-BI/*Sr-bi*, *Cyp7a1*, *Ntcp*, *Ostβ* and *Bsep* were quantified by Q-PCR using SYBR green chemistry (Fermentas, Glen Burnie, MD) and normalized to GAPDH/*Gapdh* mRNA levels. The primer sequences used in Q-PCR are presented in Table S1.

### ChIP-Seq

ChIP-seq was performed as previously reported [17]. Histograms of FXR binding to the *Sr-bi* gene in liver were generated using Affymetrix Integrated Genome Browser [41].

### ChIP-qPCR

ChIP-qPCR was performed on livers of mice treated with vehicles, GW4064 or HFD following previously described methods [17]. Briefly, fresh-frozen livers were minced and fixed in 1% formaldehyde for 15 min and then quenched with 0.125 M glycine. The cells were lysed and centrifuged. The nuclei pellet was re-suspended in nuclear lysis buffer with protease inhibitors. Nuclear extracts were sonicated to yield 500–1000 bp DNA fragments. Sonicated chromatin was aliquoted and chromatin (30 mg tissue equivalents) was used for each immunoprecipitation assay. Samples were pre-cleared with Protein agarose G-salmon sperm DNA beads (Millipore, Temecula, CA) before incubation with an IgG antibody or anti-FXR antibody (H-130x) from Santa Cruz Biotechnology (Santa Cruz, CA). Samples were incubated with prepared protein agarose G-salmon sperm DNA beads in order to extract antibody-chromatin complexes. Complexes were washed and eluted with immunoprecipitation elution buffer. DNA fragments associated with the FXR antibody were released by incubating samples in a 450 mM NaCl solution at 65°C for 5 hrs. RNA and protein were degraded by treating chromatin with RNase A and proteinase K. DNA fragments were purified by standard DNA column purification. The purified DNA fragments that were bound by FXR were analyzed by qPCR with primers amplifying four FXR binding sites located in the first intron and the downstream region of the *Sr-bi* gene. The sequences for the primers for ChIP-qPCR assay are presented in Table S1.

### Construction of plasmids for reporter gene luciferase assay

All three fragments, named as A, B and C, are located in the first intron of the *Sr-bi* gene. Fragments A and B, each of which contains a FXRRE in form of an IR1, are located from +10454 to +11066 and +21265 to +21845 relative to the transcription start site (TSS), respectively. Fragment C, containing two IR1s, is located from +27508 to +28086 relative to the TSS. All fragments were amplified from mouse genomic DNA by PCR using pairs of primers containing XhoI and BglII restriction enzyme sites, respectively (primer sequences are listed in Table S1). The PCR products, named pGL10454, pGL21265 or pGL27508, were subcloned upstream of the luciferase gene into pGL4-23 firefly luciferase vector from Promega (Madison, WI). The sequences of these constructs were confirmed by DNA sequencing and the new plasmids were named as PGL4-23-*Sr-bi* A, PGL4-23-*Sr-bi* B, and PGL4-23-*Sr-bi* C luciferase vector, respectively.

### Construction of plasmids for Site-directed mutagenesis of FXRREs

QuikChange II XL Site-Directed Mutagenesis Kit (Stratagene, La Jolla, CA) was used to generate mutations of the IR1 sites in PGL4-23-*Sr-bi* A, PGL4-23-*Sr-bi* B, and PGL4-23-*Sr-bi* C, according to the manufacturer's instruction. Primers for site-directed mutagenesis are given in Table S1. The desired mutations were verified by DNA sequencing, and the mutated plasmids were named as Mu-PGL4-23-*Sr-bi* A, Mu-PGL4-23-*Sr-bi* B, Mu-PGL4-23-*Sr-bi* C-1^st^ IR1, Mu-PGL4-23-*Sr-bi* C-2^nd^ IR1 and Mu-PGL4-23-*Sr-bi* C-(1^st^+2^nd^) IR1 luciferase vector, respectively.

### Transient transfection and luciferase reporter gene assays

Briefly, HepG2 cells were seeded in a 96-well plate and grown to 90% confluency prior to transient transfection with various pGL4-23 reporter gene constructs as well as pCMV-ICIS human FXR (Open Biosystems, Huntsville, AL), pSG5 human RXRα (Stratagene, La Jolla, CA), and pCMV-renilla luciferase vector (Promega, Madison, WI). Transient transfection was carried out according to the manufacturer's instructions using TurboFect *in vitro* transfection reagent (Fermentas, Glen Burnie, MD). Five hrs after transfection, cells were treated with 1 µM GW4064 or 0.1% DMSO as negative control. Thirty-six hrs after treatment, firefly luciferase and renilla luciferase activities were quantified using the Dual-Glo Luciferase Kit (Promega, Madison, WI) in a Synergy-II HT plate reader (Bio-Tek Instruments, Inc., Winooski, VT). The firefly luciferase activity value was normalized as a ratio to that of renilla luciferase and expressed as fold over the pGL4-23 empty vector control. The data were presented as an average of six wells and the experiments were repeated at least twice.

### Determination of cholesterol levels in mouse serum and livers

After WT and FXR-KO mice were fed either regular rodent chow or HFD for 16 weeks, serum was obtained by centrifugation of blood at 8,000×*g* using microtainer serum separator tubes (BD Biosciences, San Jose, CA) for 15 min at 4°C and stored at −80°C for analysis. Liver lipid content was also extracted as described previously [42]. Briefly, 100 mg of frozen liver tissue was homogenized in 1 ml of buffer containing 18 mM Tris, pH 7.5, 300 mM mannitol, 50 mM EGTA, and 0.1 mM phenylmethylsulfonyl fluoride. Five hundred microliters of homogenate was mixed with 4 ml of chloroform/methanol (2∶1) and incubated overnight at room temperature with occasional shaking. Subsequently, 1 ml of H_2_O was added and the solution was vortexed and subjected to centrifugation for 5 minutes at 3000×*g*. The lower lipid phase was then collected and concentrated by vacuum. The lipid pellets were dissolved in a mixture of 270 µl isopropanol and 30 µl of Triton X-100. The kit for analyzing serum and liver total cholesterol was obtained from Wako Bioproducts (Richmond, VA). The kit for analyzing serum HDL, LDL/VLDL activity was obtained from Abcam (Cambridge, MA). All measurements were performed according to the manufacturer's instructions.

### Statistical analysis

All data were presented as mean ± SD. All data were analyzed by one-way analysis of variance followed by the Student-Newman-Keuls test. P<0.05 was considered statistically significant.

## Supporting Information

Figure S1 **FXR activation in mouse livers by treatment of FXR agonists or genetic over-expression of FXR.** **A**, Induction of FXR targets *Cyp7a1*, *Ntcp*, *Ostβ and Bsep* mRNA levels in the liver following treatment with either 1% cholic acid (CA)-containing diet or GW4064 in WT and FXR-KO mice as described in the Methods. An asterisk indicates P<0.05 and double asterisks mean P<0.01 between vehicle and ligand treatment group. **B**, *Cyp7a1*, *Ntcp*, *Ostβ and Bsep* mRNA expression levels in liver of FXR-WT and FXR-Tg mice. An asterisk means P<0.05 and double asterisks indicate P<0.01 between FXR-WT and FXR-Tg group.
(TIF)

Table S1 **Primers used for Q-PCR, ChIP-qPCR, clone and mutation.**
(DOC)
