# Supplementary figures and images for: Farnesoid X Receptor Induces Murine Scavenger Receptor Class B Type I via Intron Binding
Source: PLoS One. 2012 Apr 23;7(4):e35895. doi: 10.1371/journal.pone.0035895 (PMC3335076; doi:10.1371/journal.pone.0035895)

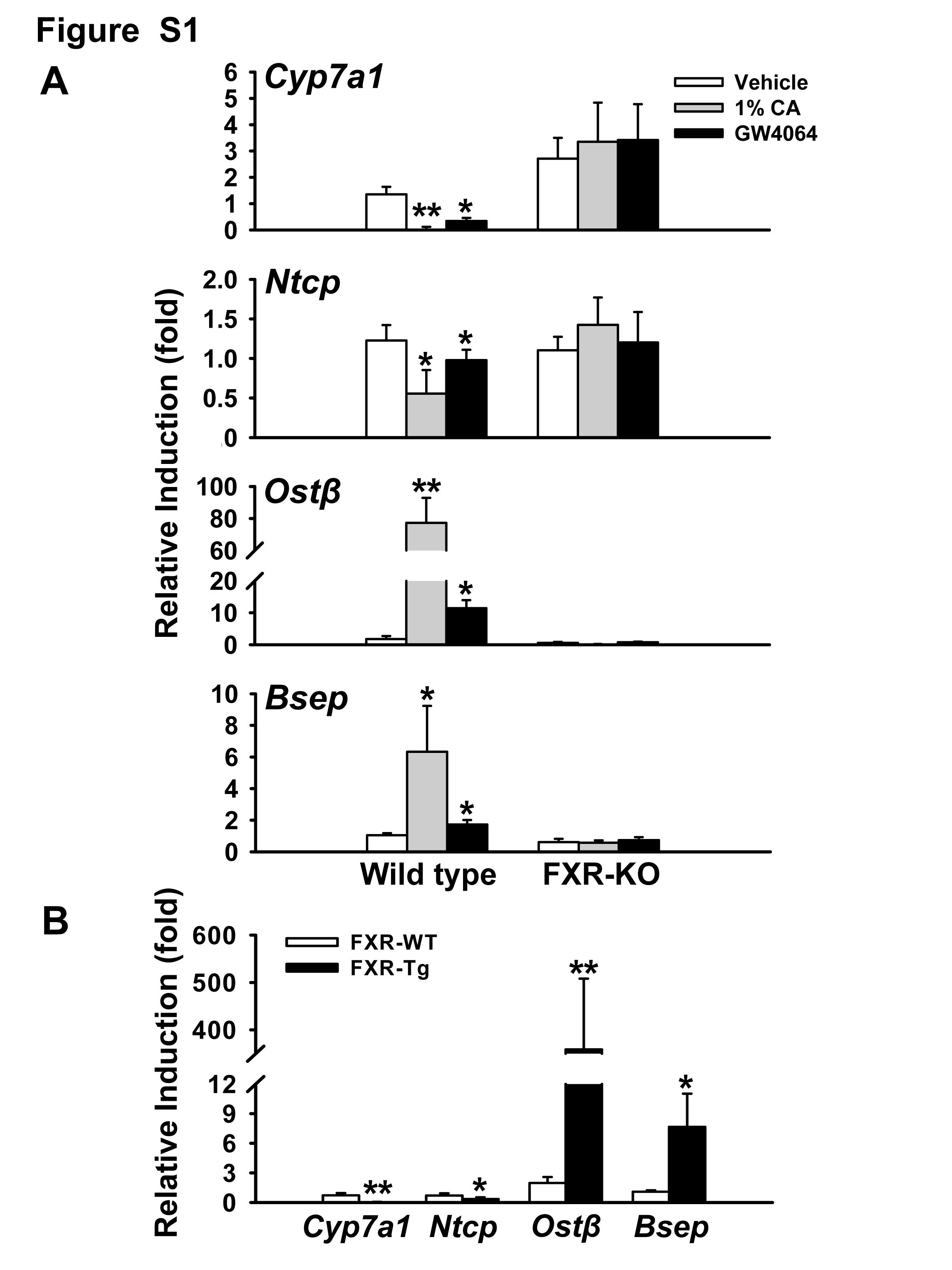

Supplement: Figure S1 — FXR activation in mouse livers by treatment of FXR agonists or genetic over-expression of FXR. A, Induction of FXR targets Cyp7a1, Ntcp, Ostβ and Bsep mRNA levels in the liver following treatment with either 1% cholic acid (CA)-containing diet or GW4064 in WT and FXR-KO mice as described in the Methods. An asterisk indicates P<0.05 and double asterisks mean P<0.01 between vehicle and ligand treatment group. B, Cyp7a1, Ntcp, Ostβ and Bsep mRNA expression levels in liver of FXR-WT and FXR-Tg mice. An asterisk means P<0.05 and double asterisks indicate P<0.01 between FXR-WT and FXR-Tg group. (TIF) [file pone.0035895.s001.tif]
