# Supplementary material for: Farnesoid X Receptor Induces Murine Scavenger Receptor Class B Type I via Intron Binding
Source: PLoS One. 2012 Apr 23;7(4):e35895. doi: 10.1371/journal.pone.0035895 (PMC3335076; doi:10.1371/journal.pone.0035895)
Supplement: Table S1 — Primers used for Q-PCR, ChIP-qPCR, clone and mutation. (DOC) [file pone.0035895.s002.doc]

| **Table S1: Primers used for Q-PCR, ChIP-qPCR, clone and mutation** | |
| --- | --- |
| **Primer Name** | **Primer Sequence: 5'-3'** |
| **Mouse *Fxr* Forward** | **CTTGATGTGCTACAAAAGCTGTG** |
| **Mouse *Fxr* Reverse** | **ACTCTCCAAGACATCAGCATCTC** |
| **Mouse *Shp* Forward** | **CGATCCTCTTCAACCCAGATG** |
| **Mouse *Shp* Reverse** | **AGGGCTCCAAGACTTCACACA** |
| **Mouse *Sr-bi* Forward** | **TCCCCATGAACTGTTCTGTGAA** |
| **Mouse *Sr-bi* Reverse** | **TGCCCGATGCCCTTGACA** |
| **Mouse *Cyp7a1* Forward** | **AGCAACTAAACAACCTGCCAGTACTA** |
| **Mouse *Cyp7a1* Reverse** | **GTCCGGATATTCAAGGATGCA** |
| **Mouse *Ntcp* Forward** | **GGCCACAGACACTGCGCT** |
| **Mouse *Ntcp* Reverse** | **AGTGAGCCTTGATCTTGCTGAACT** |
| **Mouse *Ostβ* Forward** | **GTATTTTCGTGCAGAAGATGCG** |
| **Mouse *Ostβ* Reverse** | **TTTCTGTTTGCCAGGATGCTC** |
| **Mouse *Bsep* Forward** | **ACAGAAGCAAAGGGTAGCCATC** |
| **Mouse *Bsep* Reverse** | **GGTAGCCATGTCCAGAAGCAG** |
| **Mouse *Gapdh* Forward** | **TGTGTCCGTCGTGGATCTGA** |
| **Mouse *Gapdh* Reverse** | **CCTGCTTCACCACCTTCTTGAT** |
| **Human FXR Forward** | **TGCATTGAAGTTGCTCTCAGGT** |
| **Human FXR Reverse** | **CGCCTGACTGAATTACGGACA** |
| **Human SHP Forward** | **AGCTGGAAGTGAGAGCAGATCC** |
| **Human SHP Reverse** | **AGAAGTGCGTAGAGAATGGCG** |
| **Human SR-BI Forward** | **CACCTTCAACAACAACGACACCGT** |
| **Human SR-BI Reverse** | **ATGAGCTTCAGGGTCATGGGCTTA** |
| **Human GAPDH Forward** | **GGTGGTCTCCTCTGACTTCAA** |
| **Human GAPDH Reverse** | **GTTGCTGTAGCCAAATTCGTTGT** |
| **ChIP *Sr-bi* site A Forward** | **CGCTGAGGTCAATTCAGACCCAAT** |
| **ChIP *Sr-bi* site A Reverse** | **TAAACATTTGGGAACCCAAGGCCC** |
| **ChIP *Sr-bi* site B Forward** | **AAATCACCAGTCTGTCCTGATAGCCG** |
| **ChIP *Srbi* site B Reverse** | **ACCTTTGTCTTCCCAGAGGGTCAT** |
| **ChIP *Sr-bi* site C Forward** | **CCTGGCTTCTTCCTCTATTGCCTT** |
| **ChIP *Sr-bi* site C Reverse** | **CGTAGGACAGAAGCAGCTTAAGGA** |
| **ChIP *Sr-bi* site D Forward** | **GCAAATGCATGCGCTTCTGTGAGT** |
| **ChIP *Sr-bi* site D Reverse** | **CCAACCCAAAGCTCCTTGCACATT** |
| ***Sr-bi* 10454 to 11066 Forward** | **TGCCCTCGAGTACCTTTCTGGCTT** |
| ***Sr-bi* 10454 to 11066 Reverse** | **CCCAGATCTGTTGCAGGAATGAAT** |
| ***Sr-bi* 21265 to 21845 Forward** | **TAAGCTCGAGCAGGGCACAGTCAA** |
| ***Sr-bi* 21265 to 21845 Reverse** | **ACACGAGATCTTGGTGCTGGAATA** |
| ***Sr-bi* 27508 to 28086 Forward** | **TGTGCTCGAGCCATCCTAACTGCT** |
| ***Sr-bi* 27508 to 28086 Reverse** | **ACACGAGATCTCCTATGTGTATGT** |
| **Mu *Sr-bi* site A IR1 Forward** | **GGGAGTCCCCAGGCTCAGGATTACAAGTACCTCTGTGGTGTTTGT** |
| **Mu *Sr-bi* site A IR1 Reverse** | **ACAAACACCACAGAGGTACTTGTAATCCTGAGCCTGGGGACTCCC** |
| **Mu *Sr-bi* site B IR1 Forward** | **AGTTTGAAGCCAGTCCAGATTGGTATGACGCTCTGGGAAGACAAAGGTG** |
| **Mu *Sr-bi* site B IR1 Reverse** | **CACCTTTGTCTTCCCAGAGCGTCATACCAATCTGGACTGGCTTCAAACT** |
| **Mu *Sr-bi* site C-1^st^ IR1 Forward** | **AAGGAGAAAGATCCTAGCATGCGTTCACCCCTCCCTGCTTCC** |
| **Mu *Sr-bi* site C-1^st^ IR1 Reverse** | **GGAAGCAGGGAGGGGTGAACGCATGCTAGGATCTTTCTCCTT** |
| **Mu *Sr-bi* site C-2^nd^ IR1 Forward** | **CACCAAGATGGACTGCGGCTTTTACCTGTGACCCAGATTAGCCTTCC** |
| **Mu *Sr-bi* site C-2^nd^ IR1 Reverse** | **GGAAGGCTAATCTGGGTCACAGGTAAAAGCCGCAGTCCATCTTGGTG** |
